# Characteristics of the phyllosphere microbial community and its relationship with major aroma precursors during the tobacco maturation process

**DOI:** 10.3389/fpls.2024.1346154

**Published:** 2024-05-10

**Authors:** Yixuan Shi, Yuansheng He, Yuanxian Zheng, Xixi Liu, Shuzhong Wang, Tian’e Xiong, Tao Wen, Hong Duan, Xiaolin Liao, Quanren Cui, Fuzhao Nian

**Affiliations:** ^1^ College of Tobacco Science, Yunnan Agricultural University, Kunming, Yunnan, China; ^2^ Technology and Research Center, Lincang Branch Company of Yunnan Tobacco Company, Lincang Yunnan, China; ^3^ College of Food Science and Technology, Yunnan Agricultural University, Kunming, Yunnan, China; ^4^ Tobacco Research Institute, Anhui Academy of Agricultural Sciences, Hefei, Anhui, China

**Keywords:** tobacco, phyllosphere microbiota, ecological zone, top pruning, aroma precursors

## Abstract

Numerous bacteria, fungi and other microorganisms in the tobacco phyllosphere interstellar area participate in the physiological metabolism of plants by interacting with the host. However, there is currently little research on the characteristics of tobacco phyllosphere microbial communities, and the correlation between tobacco phyllosphere microbial communities and phyllosphere factor indicators is still unknown. Therefore, high-throughput sequencing technology based on the 16S rRNA/ITS1 gene was used to explore the diversity and composition characteristics of tobacco phyllosphere bacterial and fungal communities from different maturation processes, and to identify marker genera that distinguish phyllosphere microbial communities. In this study, the correlations between tobacco phyllosphere bacterial and fungal communities and the precursors of major aroma compounds were explored. The results showed that as the tobacco plants matured, the density of glandular trichomes on the tobacco leaves gradually decreased. The surface physicochemical properties of tobacco leaves also undergo significant changes. In addition, the overall bacterial alpha diversity in the tobacco phyllosphere area increased with maturation, while the overall fungal alpha diversity decreased. The beta diversity of bacteria and fungi in the tobacco phyllosphere area also showed significant differences. Specifically, with later top pruning time, the relative abundances of *Acidisoma*, *Ralstonia*, *Bradyrhizobium*, *Alternaria* and *Talaromyces* gradually increased, while the relative abundances of *Pseudomonas*, *Filobassidium*, and *Tausonia* gradually decreased. In the bacterial community, *Acidisoma*, *Ralstonia*, *Bradyrhizobium*, and *Alternaria* were significantly positively correlated with tobacco aroma precursors, with significant negative correlations with tobacco phyllosphere trichome morphology, while *Pseudomonas* showed the opposite pattern; In the fungal community, *Filobasidium* and *Tausonia* were significantly negatively correlated with tobacco aroma precursors, and significantly positively correlated with tobacco phyllosphere trichome morphology, while *Alternaria* showed the opposite pattern. In conclusion, the microbiota (bacteria and fungi) and aroma precursors of the tobacco phyllosphere change significantly as tobacco matures. The presence of *Acidisoma*, *Ralstonia*, *Bradyrhizobium* and *Alternaria* in the phyllosphere microbiota of tobacco may be related to the aroma precursors of tobacco.

## Introduction

The phyllosphere mainly refers to the environment formed by leaves, including the surface and internal environment (Ruinen, 1961). Phyllosphere microorganisms refer to the collective term for epiphytic bacteria on the surface and endophytic bacteria inside (Marie-Agnès and Morris, 1995; Mina et al., 2020). The phyllosphere of plants is rich in biodiversity, and includes various bacteria, archaea, fungi, oomycetes, nematodes, and viruses (Wallace et al., 2018; Bashir et al., 2022).Archaea, viruses, and other microbes in the phyllosphere are poorly characterized due to the lack of related research. Therefore, bacteria and fungi are the most studied groups in plant tissues. Phyllosphere microorganisms play a very important role in the phyllosphere, as they can promote plant growth by promoting nutrient absorption, synthesizing plant hormones, and assisting plants in adapting to abiotic stress (Vorholt, 2012). Phyllosphere microorganisms can also induce systemic resistance in plants to maintain plant health through nutrient or spatial competition, the production of antibacterial metabolites, and interference with plant pathogen quorum sensing (Berg and Koskella, 2018). Chen et al. reported that when the balance of the leaf microbiota is disrupted, microbial diversity decreases, and *Proteobacteria* proliferate and inhibit *Bacillota* growth, leading to symptoms such as leaf tissue yellowing or necrosis in plants (Chen et al., 2020). In addition, leaf microorganisms can produce alkaloids, terpenes, esters, and other compounds, which affect the diversity of plant metabolites. Mucciarelli et al. reported that endophytic bacteria are beneficial for increasing terpenoids levels in the leaves of Mentha canadensis (Mucciarelli et al., 2007). Due to their long-term symbiosis and coevolution with the host plant, some endophytic fungi can produce secondary metabolites that are the same or similar to those of the host. For example, Stierle et al. isolated the endophytic fungus Taxomyces andreanae from Taxus wallichiana var. chenensis, which can produce paclitaxel (Stierle et al., 1993). Phyllosphere microorganisms have high biodiversity, complex community structures, and large biomasses (Vorholt, 2012; Laforest-Lapointe and Whitaker, 2019; Bashir et al., 2022; Sohrabi et al., 2023). The interaction between plants and their potential functions is a new research field in the interdisciplinary research of botany and microbiology (Berg et al., 2016; Das et al., 2022; Pathak et al., 2022). However, there are currently few related studies, far behind the research on rhizosphere microorganisms (Mendes et al., 2013; Qu et al., 2020; Ling et al., 2022; Liu et al., 2023). Only a few studies have focused on the stimulation of plant resistance, the control of plant pathogens, and the regulation of plant respiration by phyllosphere microorganisms, which is insufficient for understanding the interactions between phyllosphere microorganisms and plants and understanding the functions of phyllosphere microorganisms (Cappelletti et al., 2016; Gharaie et al., 2017; Zhang et al., 2023). Therefore, it is important to understand the differences in the community structure of different plant phyllosphere microorganisms and their driving mechanisms from multiple perspectives in order to understand the interactions between plants and phyllosphere microorganisms.

Tobacco aroma components are mostly generated by the degradation and conversion of aroma precursors, especially some important aroma components of tobacco (Banožić et al., 2020). Each aroma component can have one or several precursors (Wu et al., 2014). Therefore, the content of aroma precursors in tobacco is closely related to the content of aroma substances and the aroma of tobacco (Deng et al., 2010; Banožić et al., 2020). Aroma precursors generally have a relatively large molecular weight, are nonvolatile or have low volatility, and do not have obvious aromas. However, they often generate specific types of aroma components after degradation and conversion. The important and extensively studied aroma precursors of tobacco include high molecular weight terpenols, fatty acids, phenols, sugar-amino acids, and alkaloids (Yinju et al., 2018). Among them, high molecular weight terpenols are the main components of tobacco glandular trichomes and are important precursors of tobacco aroma and flavor. There are two main types of terpenols cembrenediol and labdane diterpenoids. Researchers have demonstrated that fresh tobacco leaves have a relatively high content of α-cembratrienedio, which accounts for 0.7% of the fresh weight of the leaves and 50% of the total lipid content on the leaf surface (Chang and Grunwald, 1976). Moreover, all types of cembrenediol are present in ordinary burley tobacco. Labdane compounds mainly include (+)-cis-abienol and labdane diterpenes, which are abundant in menthol cigarettes but less common in flue-cured and burley tobacco (Vontimitta et al., 2010). During the curing period of tobacco leaves, (+)-cis-abienol oxidizes and decomposes, generating a large amount of aroma substances, giving the smoke a pleasant aroma and flavor similar to those of pine wood (Guo et al., 1994; Vontimitta et al., 2010). (+)-cis-abienol is the main labdane compound in the fresh tobacco leaves of menthol cigarettes, and it is also a plant growth regulator. Fatty acids, which can be esterified with alcohols or exist in a free form, are also important aroma precursors in tobacco (Liu et al., 2022a). Higher fatty acids (palmitic acid, stearic acid, etc.) do not have a direct effect on the aroma of tobacco, but they can regulate the acidity of tobacco, make the smoke mellow, and indirectly affect the aroma of the smoke, playing a balancing role in the smoke (Kawasaki et al., 1998). Although polyphenolic compounds have very low volatility and very few directly enter smoke during combustion and inhalation, they participate in important reactions during the curing period, increasing the complexity of the aroma and improving the balance of the smoke, making them important aroma precursors in tobacco (Liu et al., 2022b). Although previous research has studied microbes in tobacco leaves and aroma precursors, there is little research on the changes and differences in the microbial community and the relationships between the microbial community and aroma precursors in the intercellular space of tobacco leaves (English et al., 1967).

Therefore, this study focused on fresh tobacco leaves and used high-throughput sequencing technology based on the 16S rRNA/ITS1 gene to sequence bacteria and fungi on the tobacco phyllosphere surface. The characteristics of the tobacco phyllosphere microbial community are elucidated from changes in the phyllosphere microbial community during the maturation process. On this basis, the correlation between the characteristics of phyllosphere bacterial and fungal communities and aroma precursors was explored. The research results not only provide a theoretical basis for subsequent related research, but also fill the gap in knowledge on the correlation between the tobacco phyllosphere microbiota and aroma precursors.

## Materials and methods

### Experimental design and sample collection

To study the changes in the microbial community of tobacco leaves during the maturation process and their relationship with tobacco aroma precursors, the representative tobacco variety Zhongyan 100 from Yiyang County, Luoyang City, Henan Province, was sampled and analyzed. In terms of botanical classification, Zhongyan 100 is a member of the Nicotiana tabacum (Solanaceae). Zhongyan 100 is a high-quality multi-resistance flue-cured tobacco variety developed by the China Tobacco Genetic and Breeding Research (Northern) Center, which hybridizes the high-quality variety NC82 with the multi-resistance complementary parent 9201. After 5 generations of backcrossing with NC82, the variety was selectively selected and cultivated using the pedigree method. This study was approved by the China Tobacco Variety Approval Committee in December 2022). The soil type of the tested field was sandy loam, and the previous season planting model was winter fallow stubble. The agronomic characteristics of the soil were as follows: pH, 7.76; organic matter, 6.03 g/kg; alkali-hydrolyzed nitrogen, 80.71 mg/kg; available phosphorus, 5.88 mg/kg; and available potassium, 123.24 mg/kg. Cultivation management followed the local quality tobacco leaf management standards. Fresh tobacco leaf samples were collected 20 d (BD20), 40 d (BD40), and 60 d (BD60) after top pruning (on days without rain or fog). In each sampling field, 5 points were selected, 5 healthy plants were chosen at each point using the five-point sampling method, and then the samples from these 5 points were merged into one sample. Fifteen 5-point sampling methods were used at each time point, and a total of 15 samples were collected.

### Tobacco phyllosphere sample processing

To preserve and restore the characteristics of fresh tobacco leaves to the greatest extent possible, a portion of the tobacco leaves collected from the field were quickly placed in 50 mL sterile centrifuge tubes, wrapped in tin foil, and flash-frozen in liquid nitrogen. The fully frozen tobacco leaf samples were then transferred to the laboratory and buried in dry ice for the extraction of phyllosphere microbial DNA. After removing the stems, another portion of the leaves was placed in self-sealing bags and transported to the laboratory using dry ice for the determination of surface secretions and leaf chemical indicators. Portions of the leaves were punched at symmetrical positions on both sides of the main vein at the widest part of the leaf using a hole puncher. Two to three circular pieces were taken from the same variety, mixed together, and fixed in Eppendorf (EP) tubes containing 2.5% glutaraldehyde for observation under a scanning electron microscope.

### Morphology of glandular trichomes in the tobacco phyllosphere

The fresh tobacco leaf samples were fixed in EP tubes containing 2.5% glutaraldehyde for 4 h, followed by fixation in 1% osmic acid for 1 h. The circular pieces were then washed three times with 0.1 mol/L phosphate buffer solution, with each wash lasting 10 min. Then, a gradient dehydration process was conducted using a solvent mixture of tert-butanol and ethanol at concentrations of 30%, 50%, 70%, and 90%, with each dehydration step lasting 5 min. After that, the samples were dehydrated twice in 100% tert-butanol, with each dehydration step lasting 5 min. Finally, the tert-butanol was placed in a refrigerator at 4°C until it solidified, and the samples were freeze-dried under vacuum. The dried samples were then glued onto sample holders using conductive adhesive and coated with gold using a HITACHI E-1010 ion sputter. The treated samples were observed and photographed for the morphology and density of glandular trichomes on the surface tobacco leaves at different varieties and for different time periods using a HITACHI SU3500 scanning electron microscope.

### Determination of aroma precursors in the tobacco phyllosphere

(1) Polyphenols: First, 0.5 g of vacuum freeze-dried tobacco leaf powder, was accurately weighed, 25 mL of 80% acetone was added, and the sample was treated with 100 Hz ultrasound at room temperature for 30 min. After centrifugation, the supernatant was collected, the residue was extracted twice via the same method, and the three supernatants. were combined. The mixture at 45°C under reduced pressure, and the precipitate was diluted with methanol to 10 mL. Filter through a 0.45 μm organic membrane to obtain the tobacco polyphenol test solution. The Folin-phenol method was used with gallic acid as a standard to create a standard curve, and the absorbance was used as the ordinate for determination (Vázquez et al., 2015).

(2) Flavonoids: First, 0.5 g of vacuum freeze-dried tobacco leaf powder was accurately weighed, 30 mL of 30% ethanol was added, and the mixture was extracted in a constant-temperature water bath at 65°C for 2 h. The hot solution was filtered through qualitative filter paper, and the filtrate was transferred to a 50 mL volumetric flask. The filter paper and residue were rinsed with 30% ethanol, and the combined filtrate was diluted with 30% ethanol to the same volume as the test solution. The aluminum nitrate colorimetric method was used with rutin as a standard to create a standard curve, and absorbance was used as the ordinate for determination (Chang et al., 2012).

(3) Alkaloids: The visible spectrophotometry method in the ELISA kit (Shanghai Lianmai Biotechnology, Double antibody sandwich method) was used for determination. Briefly, the tissue was washed with precooled PBS (0.01 M, pH=7.4), weighed, cut into small pieces, and ground with the corresponding volume of PBS (1:9) on ice. Finally, the homogenate was centrifuged at 4 °C and 5000 rpm for 10 min, and the supernatant was collected for detection according to the instructions of the ELISA kit.

(4) Soluble total sugars: First, 20 mg of vacuum freeze-dried tobacco leaf was placed in a 10 mL centrifuge tube, 4 mL of 80% ethanol was added, and the mixture was heated in a water bath at 80°C for approximately 30 min and shaken a few times during the process. The mixture was centrifuged at 5000 rpm for 10 min, and the supernatant was collected. This step was repeated three times. Subsequently, the supernatant was combined and diluted to 10 mL to determine the total soluble sugars (Zhu et al., 2012).

(5) Starch: After transferring the supernatant from the precipitate for soluble sugar determination, 2 mL of distilled water was added, and the mixture was boiled in a water bath for 15 min. Then, 1 mL of 9.2 mol/L perchloric acid solution was added, the mixture was shaken for 15 min, 2 mL of distilled water was added, and the mixture was mixed well. The mixture was centrifuged at 5000 rpm for 10 min, and the supernatant was collected. This step was repeated three times. Subsequently, the supernatant was combined and diluted to 10 mL to determine the total soluble sugars (Lustinec et al., 1983).

(6) Amino acids: First, 1 g of vacuum freeze-dried tobacco leaf powder sample was accurately weighed in a 100 mL grinding mouth triangular flask. Then, 50 mL of hydrochloric acid solution was added, and the mixture was sealed with a stopper, ultrasonicated, and filtered. Two milliliters of the filtrate was accurately removed, concentrated, and evaporated (the temperature did not exceed 60°C). One milliliter of sample diluent was added, and the mixture was shaken well. The solution was filtered through a 0.45 μm filter membrane, and the sample was measured by RP-HPLC (Lei et al., 2006).

(7) The extraction and determination of leaf surface secretions (α-cembratrienedio and β-cembrenediol) from tobacco leaves were carried out according to the methods of Marija et al (Banožić et al., 2021). Twenty circular leaf pieces with a diameter of 10 cm were cut on both sides of the main vein of the tobacco leaves. The leaf circular pieces were extracted in 500 mL of dichloromethane, each for 2 seconds, for a total of 8 times. After filtration, 1 mL of internal standard containing N-17-alkanols was added. The extract was concentrated to 50 mL using a rotary evaporator, and after derivatization, it was analyzed by GC/MS in combination with a computer. The composition of various substances in the leaf surface secretions was determined by retrieving the NIST12 spectral library. The retention time of each peak was obtained based on the peak elution time on the chromatographic flow curve of different substances, and the substances were quantified using the internal standard method.

### Phyllosphere microbiota (bacterial and fungal communities) of the tobacco plants

#### Extraction of genomic DNA from the tobacco phyllosphere microbiota

Microbial cells were collected from the leaves following the extraction method proposed by Kembel (Kembelsteven and Muellerrebecca, 2013). Briefly, 10 g of tobacco leaf tip sample was weighed and placed in a sterile triangular flask to collect microorganisms on the surface of tobacco leaves. Total DNA was extracted from the filter membrane using a Fast DNA Spin Kit (Qbiogene, Irvine, CA). The instructions of the kit were followed to dissolve the extracted DNA in 100 µL of ddH2O and store it at -20°C.

#### 16S rRNA and ITS PCR amplification of the tobacco phyllosphere microbiota

The bacterial 16S rRNA gene V4-V5 region and the fungal ITS1 region were sequenced for 45 samples using the Illumina MiSeq sequencing platform. A total of 90 sequences were obtained for bacteria and fungi. The bacterial primers used for amplification were 515F: (5’-AACMGGATTAGATACCCKG-3’) and 907R: (5’-ACGTCATCCCCACCTTCC-3’). The fungal primers used were ITS5F: GGAAGTAAAAGTCGTAACAAGG and ITS1R: GCTGCGTTCTTCATCGATGC. The amplification program was as follows: predenaturation at 98 °C for 5 min, followed by 25 cycles (denaturation at 98 °C for 30 s, annealing at 53 °C for 30 s, and extension at 72 °C for 45 s), extension at 72 °C for 10 min, and storage at 4 °C. The PCR mixture was 5× Trans Start Fast Pfu Buffer (5 μL), 2.5 mmol/L dNTPs (2 μL), forward primer (10 μMol/L) (1 μL), reverse primer (10 μMol/L) (1 μL), fast PFU DNA polymerase (5 U/μL) (0.25 μL), template DNA (1 μL), and ddH2O (14.75 μL). The quality of the library was evaluated on a Qubit@2.0 fluorometer (Thermo Scientific) and an Agilent Bioanalyzer 2100 system. The PCR products were detected by 2% agarose gel electrophoresis, and the PCR amplification recovery products were quantified using fluorescence. According to the fluorescence quantification results and the sequencing volume requirements of each sample, the purified products of each sample were mixed in the corresponding proportions for purification before loading and analysis by a sequencer (Redford et al., 2010). The sequencing procedure for this study was completed by Shanghai Paisenno Biotechnology Co., Ltd.

### Bioinformatics analysis

After using the Illumina MiSeq sequencing platform for paired-end sequencing, the obtained raw reads were subjected to quality control, and low-quality sequences (average quality of 50 consecutive bases <25, sequence length <50 bp, and 1 or more ambiguous bases) were discarded. The DADA2 (divisive amplicon denoising algorithm 2) method (DADA2: high-resolution sample inference from Illumina amplicon data) was used to perform primer trimming, quality filtering, denoising, merging, and chimera removal on the paired-end data to obtain clean reads.

First, QIIME2 software (Bolyen et al., 2019) (Quantitative Insights Into Microbial Ecology, v1.8.0) was used to identify ambiguous sequences. Then, the DADA2 method in the QIIME2 software was used to quality control, denoising, stitching, and de chimeric sequences. Sequences derived from chloroplast and mitochondria sources were also excluded to obtain high-quality sequences for analysis in this study. The UCLUST sequence alignment tool in QIIME2 software was used to cluster and assign ASVs based on 100% sequence similarity. The representative sequence of each ASV was compared with the template sequence in the corresponding database to determine its taxonomic status and obtain taxonomic information. The Greengenes and Silva databases were used for bacterial analysis, while the UNITE and Silva databases were used for fungal analysis. The QIIME2 software was used to calculate alpha diversity indices. Canoco software was used to perform principal coordinate analysis (PCoA) to investigate the impact of environmental factors on community structure. The random forest algorithm in QIIME2 software, along with nested cross-validation, was used to identify indicator species of phyllosphere microbial communities. The GeneCloud platform was used to analyze the correlations between phyllosphere microbial community structure, phyllosphere physicochemical properties, and major aroma precursors using the Spearman algorithm. Microsoft Excel 2016 and SPSS 26.0 software were used to perform ANOVA and Duncan’s *post hoc* analysis to assess the significance of differences among phyllosphere microbial diversity indices, major aroma precursors in tobacco leaves, leaf surface exudates, and phyllosphere physicochemical properties. A histogram was used to determine whether the data follows a normal distribution. If the data follows a normal distribution, perform a parametric test (one-way ANOVA analysis), with data represented as mean ± standard deviation (SD). If the data does not follow a normal distribution, use a non-parametric test (Friedman test analysis), with data presented as median [25% quantile (Q1), 75% quantile (Q3)]. Differences with *P* < 0.05 were considered to indicate statistical significance.

### Correlation analysis

Correlation analysis between the indicator bacteria and fungi and the morphology of tobacco phyllosphere trichomes and aroma precursors was conducted using GeneCloud tools, which are free online platforms for data analysis (https://www.genescloud.cn).

### Functional potential prediction

The Phylogenetic Investigation of Communities by Reconstruction of Unobserved States (PICRUSt2) (Douglas et al., 2020), available at https://github.com/picrust/picrust2/wiki, was used to predict the functional abundance of bacterial or fungal samples (Gavin M. Douglas, et al., print). In short, based on the full-length sequence of the 16S rRNA gene in the tested microbial genome, infer the gene functional profile of their common ancestor. Infer gene function profiles of other untested species in the Greengenes database and construct gene function prediction profiles for the entire lineage of archaea and bacteria domains. Finally, the composition of the microbial community obtained from sequencing is mapped to the database to predict the metabolic function of the microbial community.

### Statistical analysis

All the data were analyzed with GraphPad Prism 8.0 (GraphPad Software, San Diego, Canada). A histogram and Shapiro-Wilk analysis were used to determine whether the data follows a normal distribution. If the data follows a normal distribution, perform a parametric test (one-way ANOVA analysis), with data represented as mean ± standard deviation (SD). If the data does not follow a normal distribution, use a non-parametric test (Friedman test analysis), with data presented as median [25% quantile (Q1), 75% quantile (Q3)]. * indicates a significant difference, *P* < 0.05; ** indicates a very significant difference, *P* < 0.01; *** indicates an extremely significant difference, *P* < 0.001; NS indicates that there is no significant difference between the data, *P* > 0.05.

## Results

### Morphology of tobacco phyllosphere glandular trichomes during the ripening process

**Figure 1:**
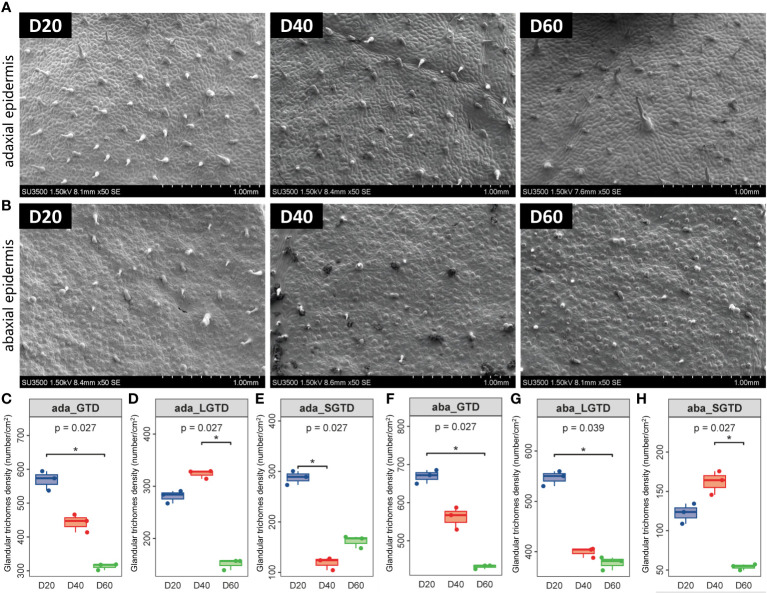
Morphology of tobacco phyllosphere glandular trichomes. **(A)** Morphological structure of glandular trichomes on the upper epidermis of the tobacco phyllosphere. **(B)** Morphological structure of glandular trichomes on the upper epidermis of the tobacco phyllosphere. **(C–E)** Statistical analysis of the morphology and structure of glandular trichomes on the upper epidermis of tobacco phyllosphere. **(F–H)** Statistical analysis of the morphology and structure of glandular trichomes on the under epidermis of tobacco phyllosphere. * represents *P*<0.05, indicating a significant difference.

Since tobacco glandular trichomes occur on more primitive epidermal cells, the overall density of glandular trichomes on tobacco leaves is greater. As the leaves develop and the leaf area expands, the density of glandular trichomes on the tobacco leaf surface decreases (*P* < 0.05) (**Figures 1A–C, F**). Specifically, the density of long-stalked glandular trichomes on the upper epidermis of tobacco leaves increased and then decreased with increasing top pruning time, while the density of long-stalked glandular trichomes on the lower epidermis gradually decreased with increasing top pruning time (*P* < 0.05) (**Figures 1D, G**). The density of short-stalked glandular trichomes on the upper epidermis of tobacco leaves gradually decreased with later top pruning times, while the density of short-stalked glandular trichomes on the lower epidermis increased and then decreased with later top pruning times (*P* < 0.05) (**Figures 1E, H**).

### Aroma precursors in the tobacco phyllosphere during the ripening process

**Figure 2:**
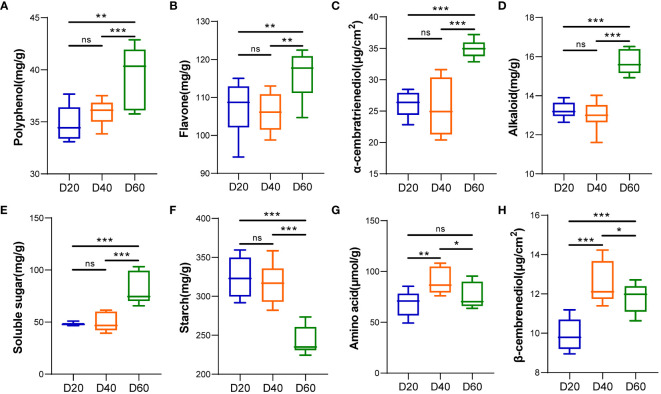
Analysis of tobacco phyllosphere surface secretions (aroma precursor substances). **(A)** Polyphenols. **(B)** Flavonoids. **(C)** α-cembratrienedio. **(D)** Alkaloids. **(E)** Soluble sugars. **(F)** Starch. **(G)** Amino acids. **(H)** β-cembrenediol. * represents *P* < 0.05, indicating a significant difference. ** represents *P* < 0.01, indicating an very significant difference. *** represents *P* < 0.001, indicating an extremely significant difference. ns represents *P* > 0.05, indicating a no significant difference.

The contents of polyphenols, flavonoids, α-cembratrienedio, alkaloids, and soluble sugars in tobacco leaf exudates peaked 60 d after top pruning and were significantly greater than those 20 and 40 d after top pruning (*P* < 0.01 or *P* < 0.001) (**Figures 2A–E**). The contents of amino acids and β-cembrenediol in tobacco leaf exudates significantly decreased with increasing pruning time (*P* < 0.05, *P* < 0.01 or *P* < 0.001) (**Figures 2G, H**). The starch content in tobacco leaf exudates gradually decreased with increasing top pruning time and reached its lowest level 60 d after top pruning (*P* < 0.001) (**Figure 2F**).

### Alpha diversity of bacterial and fungal communities in the phyllosphere area during the ripening process of tobacco

**Figure 3:**
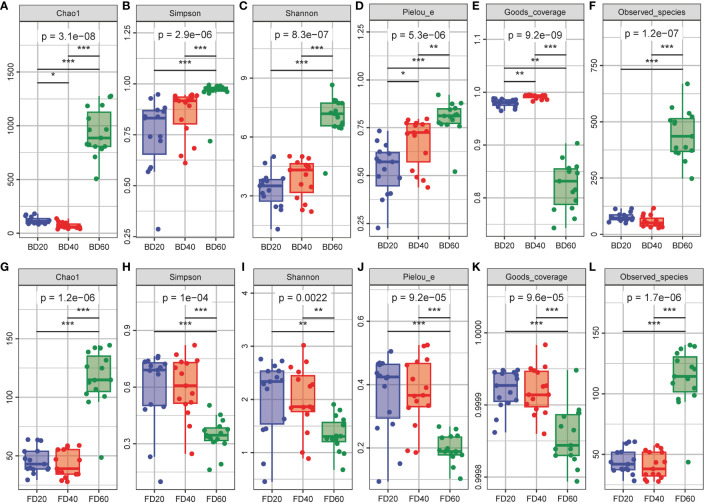
Alpha diversity of microbial community in tobacco phyllosphere. **(A–F)** Alpha diversity of bacterial community in tobacco phyllosphere. **(G–L)** Alpha diversity of fungal community in tobacco phyllosphere. * represents *P* < 0.05, indicating a significant difference. ** represents *P* < 0.01, indicating an very significant difference. *** represents *P* < 0.001, indicating an extremely significant difference.

The alpha diversity of the bacterial community in the tobacco phyllosphere gradually increased according to Simpson’s index, Shannon’s index, and Pielou’s evenness index with the number of top pruned tobacco leaves (*P* < 0.05, *P* < 0.01 or *P* < 0.001, respectively) (**Figures 3B–D**). The Chao1 index and Observed_species index first decreased and then increased with increasing top pruning time (*P* < 0.05, *P* < 0.01 or *P* < 0.001) (**Figures 3A, F**), while Good’s coverage index tended to first increase and then decrease with increasing top pruning time on tobacco leaves (*P* < 0.01 or *P* < 0.001) (**Figure 3E**). The alpha diversity of the fungal community in the tobacco phyllosphere did not significantly differ between 20 and 40 d after top pruning (*P* > 0.05), while the Chao1 and Observed_species indices of the fungal communities were significantly greater at 60 d after top pruning than at 20 and 40 d after top pruning (*P* < 0.001) (**Figures 3G, L**). The Simpson’s index, Shannon’s index, Pielou’s evenness index, and Good’s coverage index of the fungal community were also significantly greater at 60 d after top pruning than at 20 and 40 d after top pruning (*P* < 0.01 or *P* < 0.001) (**Figures 3H–K**).

### Beta diversity of bacterial and fungal communities in the phyllosphere area during the ripening process of tobacco

**Figure 4:**
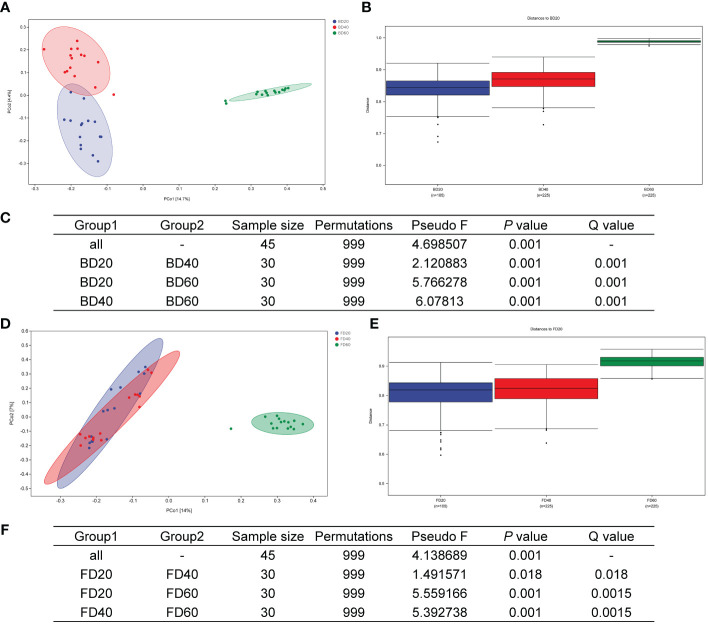
Beta diversity of microbial community in tobacco phyllosphere. **(A)** PCoA analysis of bacterial communities. **(B)** Analysis of differences between groups of bacterial communities (Figure). **(C)** Analysis of differences between groups of bacterial communities (Table). **(D)** PCoA analysis of fungal communities. **(E)** Analysis of differences between groups of fungal communities (Figure). **(F)** Analysis of differences between groups of fungal communities (Table).

The PCoA results of the bacterial community in the tobacco phyllosphere showed that there were significant differences within the community at 20 and 40 d after top pruning, while the differences within the community were relatively small at 60 d after top pruning. Moreover, as the time after top pruning increased, the differences between the groups also increased (**Figures 4A–C**). The PCoA results of the fungal community in the tobacco phyllosphere showed that there were significant differences within the community at 20 and 40 d after top pruning, while the differences within the community were relatively small at 60 d after top pruning. At the same time, the differences between the groups were relatively small at 20 and 40 d after top pruning, but they increased compared to those at 60 d after top pruning (**Figures 4D–F**).

### Composition of bacterial and fungal communities in the phyllosphere area during the ripening process of tobacco

**Figure 5:**
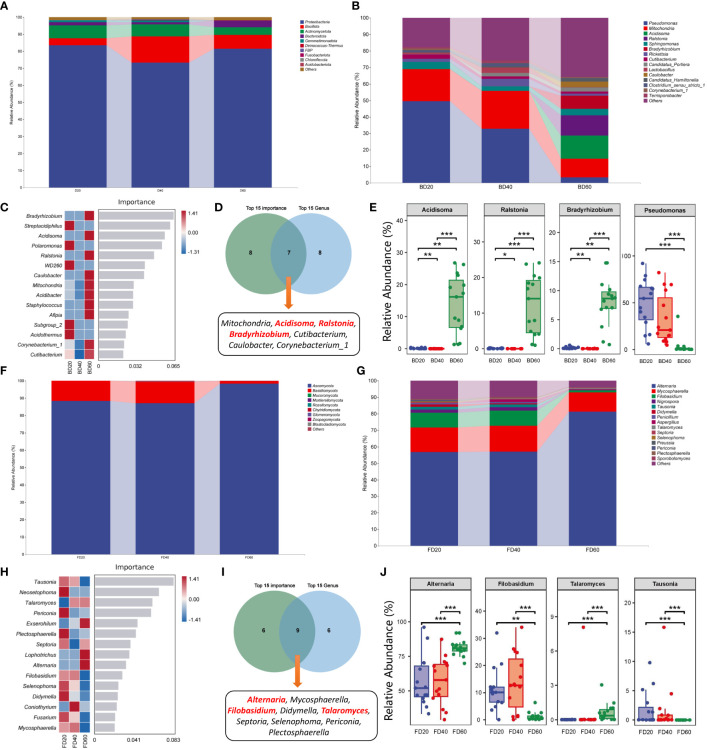
Species composition of microbial community in tobacco phyllosphere. **(A)** Species composition of bacterial communities at the phylum level. **(B)** Species composition of bacterial communities at the genus level. **(C)** Top 15 important genera of bacterial communities. **(D)** Interaction analysis of the top 15 bacteria in relative abundance and the top 15 bacteria in importance. **(E)** Differences in core bacteria during the maturation process of tobacco. **(F)** Species composition of fungal communities at the phylum level. **(G)** Species composition of fungal communities at the genus level. **(H)** Top 15 important genera of fungal communities. **(I)** Interaction analysis of the top 15 fungi in relative abundance and the top 15 fungi in importance. **(J)** Differences in core fungi during the maturation process of tobacco. * represents *P* < 0.05, indicating a significant difference. ** represents *P* < 0.01, indicating an very significant difference. *** represents *P* < 0.001, indicating an extremely significant difference.

The results of the composition of phyllosphere bacterial communities in tobacco leaves showed that at the phylum level, the relative abundance of *Bacillota* increased 40 d after top pruning, while the relative abundance of *Proteobacteria* decreased at 20 and 60 d after top pruning. However, the relative abundances of *Bacillota* and *Proteobacteria* returned to 20 d after top pruning 60 d later (**Figure 5A**). At the genus level, the relative abundance of *Pseudomonas* gradually decreased with increasing top pruning time, while the relative abundances of *Acidisoma*, *Ralstonia*, and *Bradyrhizobium* did not change at 20 and 40 days after top pruning but did increase at 60 days after top pruning (**Figure 5B**). Moreover, the results of random forest analysis further revealed that *Bradyrhizobium*, *Streptacidiphilus*, *Acidisoma*, *Polaromonas* and *Ralstonia* were the genera associated with differences among the three groups (**Figure 5C**). Furthermore, Venn analysis of the 15 most important genera and the 15 most abundant genera revealed that *Mitochondria*, *Acidisoma*, *Ralstonia*, *Bradyrhizobium*, *Cutibacterium*, *Caulobacter*, and *Corynebacterium_1* were common genera (**Figure 5D**). Moreover, the relative abundances of *Acidisoma*, *Ralstonia* and *Bradyrhizobium* in the intercellular space of tobacco leaves were significantly greater at 60 d after top pruning than at 20 d and 40 d after top pruning (*P* < 0.01 or *P* < 0.001). On the other hand, the relative abundance of *Pseudomonas* was significantly lower at 60 d after top pruning than at 20 d and 40 d after top pruning (*P* < 0.001) (**Figure 5E**).

The analysis of the composition of the intercellular fungal communities in tobacco leaves revealed that at the phylum level, there was no significant change in the fungal composition at 20 and 40 d after top pruning, while the relative abundance of Ascomycota was greater than that at 20 and 40 d after top pruning at 60 d after top pruning, and the relative abundance of Basidiomycota was less than that at 20 and 40 d after top pruning. At 60 d after top pruning, the relative abundance of *Bacillota* and Proteobacteria returned to the level observed at 20 d after top pruning (**Figure 5F**). At the genus level, there was no significant change in the fungal composition at 20 and 40 d after top pruning, while the relative abundance of *Alternaria* was greater than that at 20 and 40 d after top pruning at 60 d after top pruning, and the relative abundances of *Filobasidium*, *Nigrospora*, and *Tausonia* were less than those at 20 and 40 d after top pruning (**Figure 5G**). Moreover, the results of random forest analysis further revealed that *Tausonia*, *Neosetophoma*, *Talaromyces*, *Periconia*, and *Exserohilum* were marker genera of differences among the three groups (**Figure 5H**). Furthermore, Venn analysis of the 15 most important genera and the 15 most abundant genera revealed that *Alternaria*, *Mycosphaerella*, *Filobasidium*, *Didymella*, *Talaromyces*, *Septoria*, *Selenophoma*, and *Periconia* were common genera (**Figure 5I**). Moreover, the relative abundances of *Alternaria* and *Talaromyces* in the intercellular space of tobacco leaves were significantly greater than those at 20 and 40 d after top pruning at 60 d after top pruning (*P* < 0.01 or *P* < 0.001), while the relative abundances of *Filobasidium* and *Tausonia* were significantly lower than those at 20 and 40 d after top pruning (*P* < 0.001) (**Figure 5J**).

### Correlation analysis

**Figure 6:**
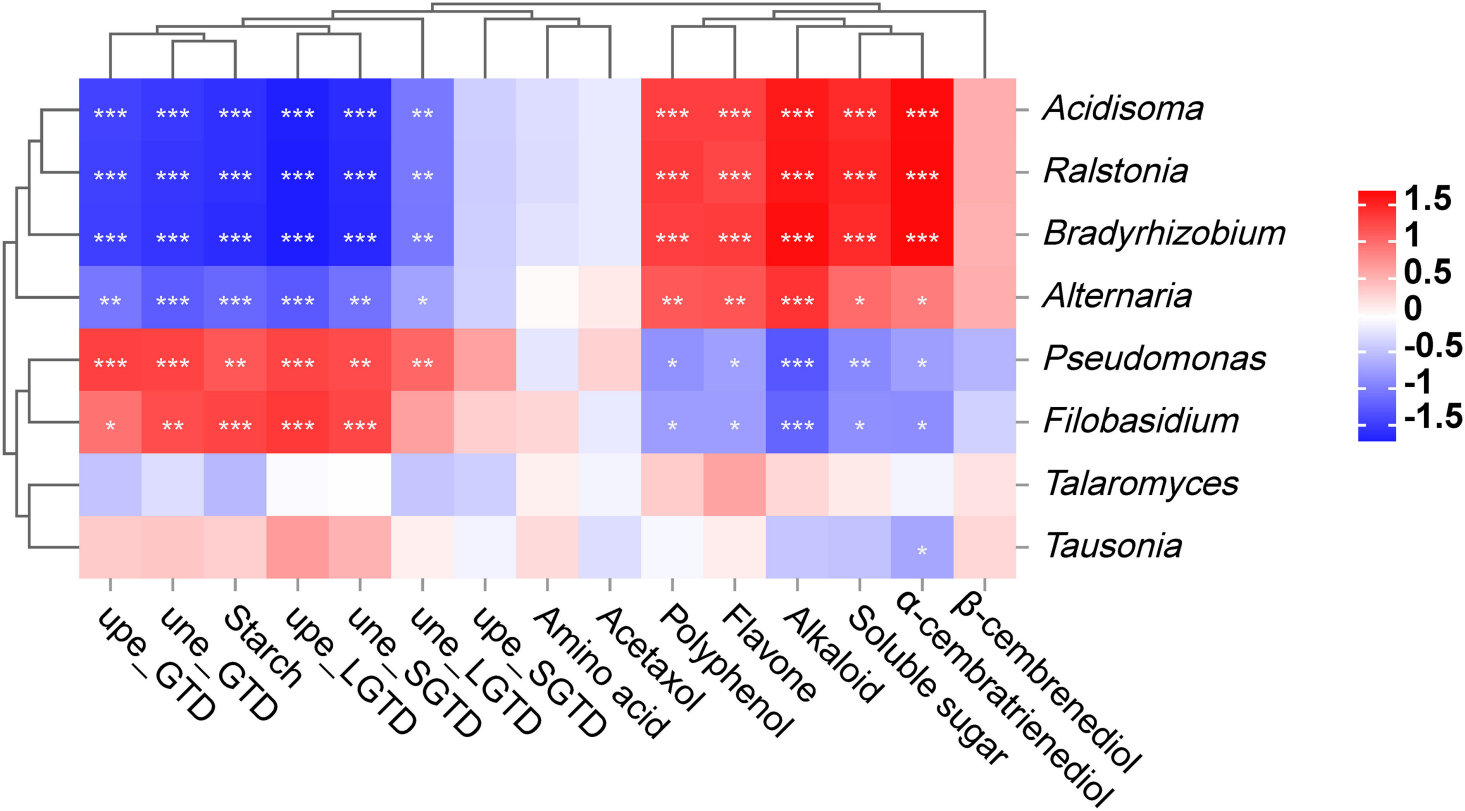
Correlation analysis between the core microbiota of tobacco phyllosphere and the morphology and secretion (aroma precursors) of tobacco phyllosphere. * represents *P* < 0.05, indicating a significant difference. ** represents *P* < 0.01, indicating an very significant difference. *** represents *P* < 0.001, indicating an extremely significant difference.

The association analysis results showed that the bacterial communities *Acidisoma*, *Ralstonia* and *Bradyrhizobium* were significantly positively correlated with tobacco aroma precursors (polyphenols, flavonoids, alkaloids, soluble sugars, and α-cembratrienedio), with significant negative correlations with tobacco leaf glandular trichome morphology (ada_GTD, aba_GTD, ada_LGTD, aba_SGTD, and aba_LGTD) (*P* < 0.05 or *P* < 0.01 or *P* < 0.001), while *Pseudomonas* showed the opposite pattern. In the fungal communities, Filobasidium and Tausonia were significantly negatively correlated with tobacco aroma precursors (polyphenols, flavonoids, alkaloids, soluble sugars, and α-cembratrienedio) and significantly positively correlated with tobacco leaf glandular trichome morphology (ada_GTD, aba_GTD, ada_LGTD, aba_SGTD, and aba_LGTD) (*P* < 0.05 or *P* < 0.01 or *P* < 0.001), while Alternaria showed the opposite pattern (**Figure 6**). Correlation analysis between other nonsignificantly different microorganisms and tobacco glandular trichomes and aroma precursors revealed that *Caulobacter* was negatively correlated with tobacco glandular trichome density but positively correlated with aroma precursors (**Supplementary Figure S1**). *Nigrospora* was positively correlated with the density of tobacco glandular trichomes and negatively correlated with aroma precursors. In addition, the correlation analysis between bacteria and fungi revealed a negative overall correlation trend (**Supplementary Figure S2**).

### Functional potential prediction results

**Figure 7:**
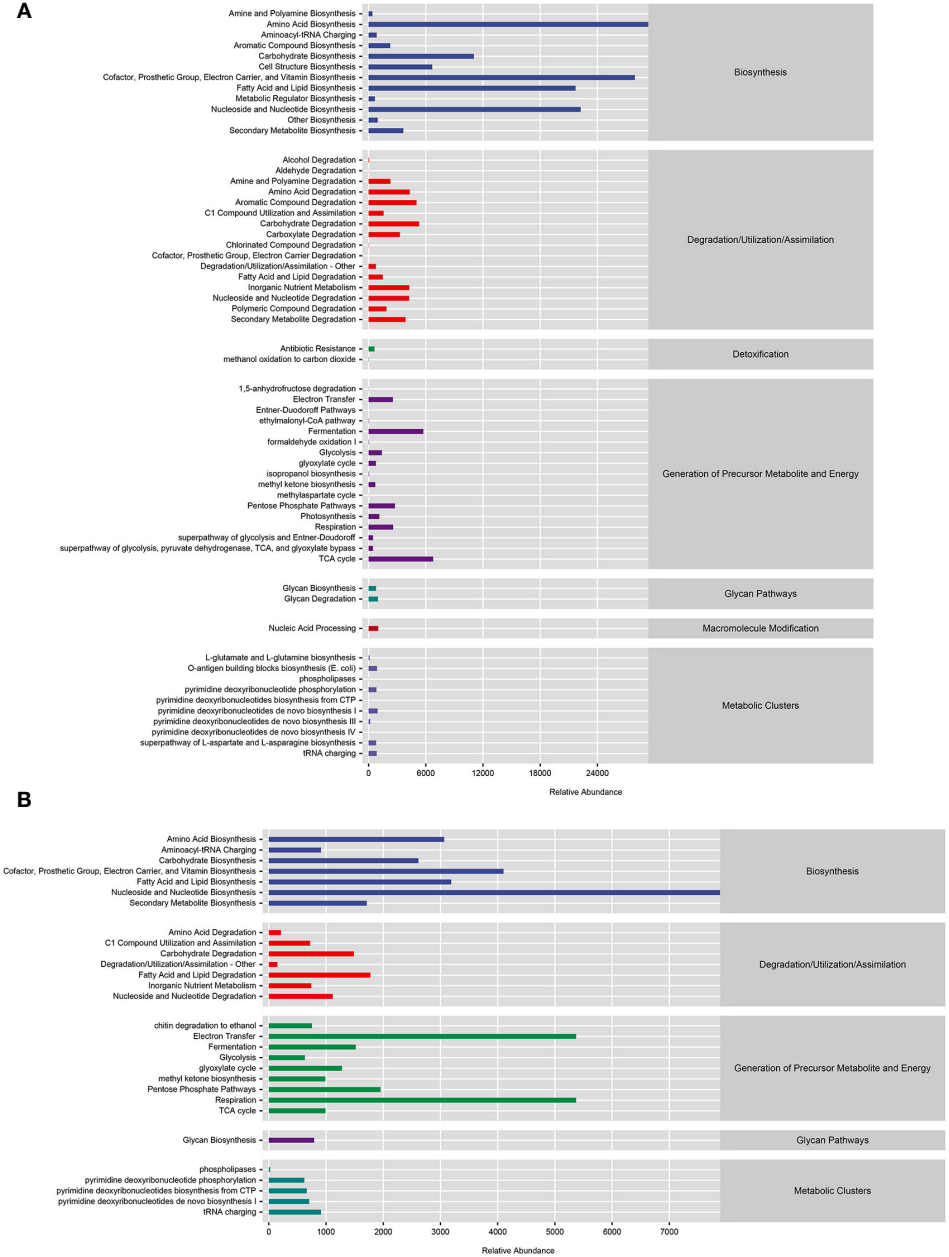
Functional prediction of microbial communities. **(A)** Functional prediction of MetaCyc metabolic pathway in bacterial community. **(B)** Functional prediction of MetaCyc metabolic pathways in fungal communities.

The PICRUSt2 analysis results showed that bacteria mainly participated in amino acid biosynthesis, cofactor, repair group, electron carrier, and vitamin biosynthesis, as well as metabolic pathways such as fatty acid and lipid biosynthesis (**Figure 7A**). Fungi mainly participated in metabolic pathways such as nucleoside and nucleotide biosynthesis, electron transfer, and respiration, as well as some amino acid biosynthesis cofactors, repair groups, electron carriers, vitamin biosynthesis, and fatty acid and lipid biosynthesis (**Figure 7B**).

## Discussion

During the ripening process of tobacco, colloidal secretions, which are precursors of many aroma substances and are closely related to the production of aroma substances in tobacco leaves, are produced on the surface of leaves (Yue et al., 2002). The tobacco phyllosphere microbiota coexists in the same growth environment as tobacco leaf surface glandular trichomes and tobacco leaf surface aroma precursors. However, there is currently little research on the characteristics of the tobacco phyllosphere microbiota community during the maturation process, and the phyllosphere factor indicators that affect changes in the tobacco phyllosphere microbiota community are not yet known.

Tobacco leaf glandular trichomes are specialized structures of leaf epidermal cells that can specifically synthesize and secrete various bioactive substances such as diterpenoids, sucrose esters, and aroma precursors, which have a positive impact on tobacco leaf quality (Keene and Wagner, 1985; Tooker et al., 2010; Huchelmann et al., 2017). Twenty days after top pruning, the cytoplasm of tobacco leaves is rich, with more contents, and the stem cells are full and plump, with robust glandular trichomes, indicating a strong secretion function of glandular trichomes at this time. Forty days after top pruning, the long-stalked glandular trichomes on the tobacco leaves exhibited significant shedding, and some of the cells became dry and shriveled, with the stem cells exhibiting a concave shape. At this time, the secretion function of glandular trichomes is weakened. At 60 d after top pruning, long-stalked glandular trichomes had shed significantly, while short-stalked glandular trichomes were more prominent and had a more complete morphology. The overall number of glandular trichomes in tobacco leaves showed a decreasing trend in the three stages after top pruning. Long-stalked glandular trichomes first appear in the juvenile stage, and at the mature stage, the glandular head breaks and releases secretions. In terms of quantity, long-stalked glandular trichomes are the main type of glandular trichome, which is consistent with the results of this study. Court suggested that varieties with high glandular trichome density also have greater glandular trichome secretion content (Shishehian et al., 2023). However, Nielsen et al. suggested that the extent of glandular trichome secretion may depend more strongly on the secretion ability of glandular trichomes and is not closely related to glandular trichome density (Nielsen and Severson, 1990). The secretion content of glandular trichomes may be influenced by multiple factors, and glandular trichome density is likely the main factor affecting the secretion content of leaf glandular trichomes. Research has shown that the density of long-stalked glandular trichomes in tobacco first increases and then decreases with increasing growth period. This may be due to differences in fertility conditions during the planting process. The mechanism by which insufficient fertility affects the development of tobacco glandular trichomes in the later stage of tobacco growth needs further research (Lin et al., 2014; Yajie et al., 2015). In addition, β-cembrenediol is not only the main product of tobacco glandular trichomes but also a precursor to the aroma of tobacco. The higher its content is, the richer the aroma of tobacco leaves (Han et al., 2013). This study showed that under the climatic conditions of the experimental area, the content of cyclodextrin in tobacco tended to first increase and then decrease with time, which is consistent with the results of Lu et al. (Leng and Lu, 2014). Moreover, the change in the content of β-cembrenediol in flue-cured tobacco is consistent with the change in the density of long-stalked glandular trichomes, and the surface cypermethritol may be closely related to the long-stalked glandular trichomes of flue-cured tobacco.

The physical and chemical properties of tobacco leaves are different, and the type and environment of the phyllosphere microbiota can lead to differences in the density of glandular trichomes on tobacco leaves. Therefore, this study further analyzed the relationships among the phyllosphere microbiota, phyllosphere physical and chemical properties, and glandular trichome traits in tobacco leaves. The results of the microbial diversity analysis showed that there was a diversity of microorganisms in the phyllosphere area during the ripening process of tobacco. Overall, the diversity of bacteria in the phyllosphere area was greater than that of fungi, which is consistent with the findings of previous study (Pugh et al., 1978). Gao et al. indicated that in the early stages of nutrient growth, bacterial α and β diversity often decreases, and these findings may explain to some extent why the initial phyllosphere microbiota had difficulty capturing new host plants during colonization, which subsequently modified them. As tobacco leaves grow, the increase in diversity and species richness may play a role in the high functional redundancy within the microbiome, enabling it to cope with complex environmental changes and quickly recover from stress (Gao et al., 2023). The opposite trend was observed for the Pielou, Shannon, and Simpson indices for the fungal and bacterial communities over time. The reason for trend may be that, as tobacco grows and develops, more nutrients are utilized by bacteria. Moreover, bacteria may have a greater ability to compete with fungi for nutrients. The association analysis between bacteria and fungi also supports this point. In addition, with increasing top pruning time, the relative abundances of *Acidisoma*, *Ralstonia*, and *Bradyrhizobium* in the bacterial community gradually increased, while the relative abundance of *Pseudomonas* gradually decreased. Similarly, the relative abundances of *Alternaria* and *Talaromyces* in the fungal community gradually increased, while the relative abundances of *Filobassidium* and *Tausonia* gradually decreased. Studies have shown that the abundance of *Pseudomonas* in susceptible tobacco leaves increases abnormally, which in turn affects the diversity of the microbial community in tobacco leaves. In this study, as the top pruning time progressed, the tobacco gradually matured, and its ability to resist pathogens gradually increased, which may be the reason for the decrease in *Pseudomonas* colonization.

Furthermore, by analyzing the correlations between phyllosphere boundary bacteria and fungi during the ripening process of tobacco and phyllosphere physiological indicators, it was found that there was a strong correlation between bacteria or fungi such as *Acidisoma*, *Ralstonia*, *Bradyrhizobium*, *Alternaria*, *Pseudomonas*, *Talaromyces*, *Filobassidium*, and *Tausonia* and the physicochemical properties of tobacco leaves. Plant phyllosphere fungi are closely related to plants, and their community structure and diversity are the result of interactions among plants, microorganisms, and other environmental factors (Chao-Jiang and Shi-Qing, 2017). The functional prediction results of PICRUST2 showed that microorganisms are mainly involved in the biosynthesis of amino acids, vitamins, and fatty acids. English et al. reported that tobacco leaves inoculated with a mixture of Bacillus subtilis and Bacillus ring-shaped bacteria can quickly produce a pleasant aroma. Further analysis revealed that with the growth of microorganisms, the total sugar and reducing sugar contents in tobacco leaves decrease (English et al., 1967). Microbial degradation of macromolecular substances in tobacco, such as starch, protein, pectin, etc., can reduce the production of harmful substances, such as nicotine and tar, which is beneficial for improving the quality of tobacco by producing small molecular substances, increasing the content of aroma components in tobacco, and improving the quality of tobacco (Chen et al., 2008). These findings indicate that microorganisms are the main factor driving the aroma precursors of tobacco. Hunter et al. reported that differences in the phyllosphere bacterial community in lettuce are closely related to phyllosphere morphological parameters, soluble carbohydrates, water content, and phyllosphere exposure, while the sugar and organic acid contents in tomato fruits are greater than those in stems and leaves. *Bacteroides thetaiotaomicron* is mainly present in the flesh, while *Rosenbergiella nectaria* is abundant in the peel (Hunter et al., 2010). These bacteria can promote the degradation of carbohydrates during tomato fruit development and maturation (Zhao et al., 2016). The differences in physiological indices among different plant parts and in the nutritional preferences of phyllosphere microorganisms may be one of the reasons for the differences in correlations between phyllosphere microorganisms and physiological indicators. Yadav et al. evaluated the impact of different phyllosphere characteristics on the size of phyllosphere bacterial communities in 8 perennial trees (Yadav et al., 2005). The results showed that the size of phyllosphere bacterial populations was positively correlated with the density of secretory and non-secretory glandular trichomes, and negatively correlated with leaf thickness, mesophyll thickness, and phyllosphere dorsal epidermis thickness. The surface exudates of fresh tobacco leaves are positively correlated with the density of secretory and non-secretory glandular trichomes (Yadav et al., 2005), and there is a certain correlation between the phyllosphere microbiota and tobacco surface exudates.

## Conclusion

In this study, as tobacco matured, the bacterial richness in the phyllosphere of tobacco gradually increased, and the fungal richness gradually decreased, with a main, negative correlation between the two parameters. Both bacteria and fungi are involved in the biosynthesis of amino acids, vitamins, and lipids, which may be important factors driving the precursor substances of tobacco aroma. The presence of *Acidisoma*, *Ralstonia*, *Bradyrhizobium* and *Alternaria* in the phyllosphere microbiota of tobacco may be related to the aroma precursors of tobacco. The abundances of *Pseudomonas* and *Filobassidium* may be correlated with the density of tobacco glandular trichomes. These microorganisms may have important potential in improving the quality of tobacco.

## Data availability statement

The datasets presented in this study can be found in online repositories. The names of the repository/repositories and accession number(s) can be found below: https://www.ncbi.nlm.nih.gov/genbank/, PRJNA1036814, https://www.ncbi.nlm.nih.gov/genbank/, PRJNA1036808.

## Author contributions

YS: Writing – original draft. YH: Writing – original draft. YZ: Methodology, Writing – review & editing. XXL: Visualization, Writing – review & editing. SW: Writing – review & editing. T’EX: Writing – review & editing. TW: Data curation, Writing – review & editing. HD: Data curation, Writing – review & editing. XLL: Writing – review & editing. QC: Writing – review & editing. FN: Funding acquisition, Resources, Supervision, Writing – review & editing.
