# Supplementary figures and images for: Characteristics of the phyllosphere microbial community and its relationship with major aroma precursors during the tobacco maturation process
Source: Front Plant Sci. 2024 May 10;15:1346154. doi: 10.3389/fpls.2024.1346154 (PMC11116568; doi:10.3389/fpls.2024.1346154)

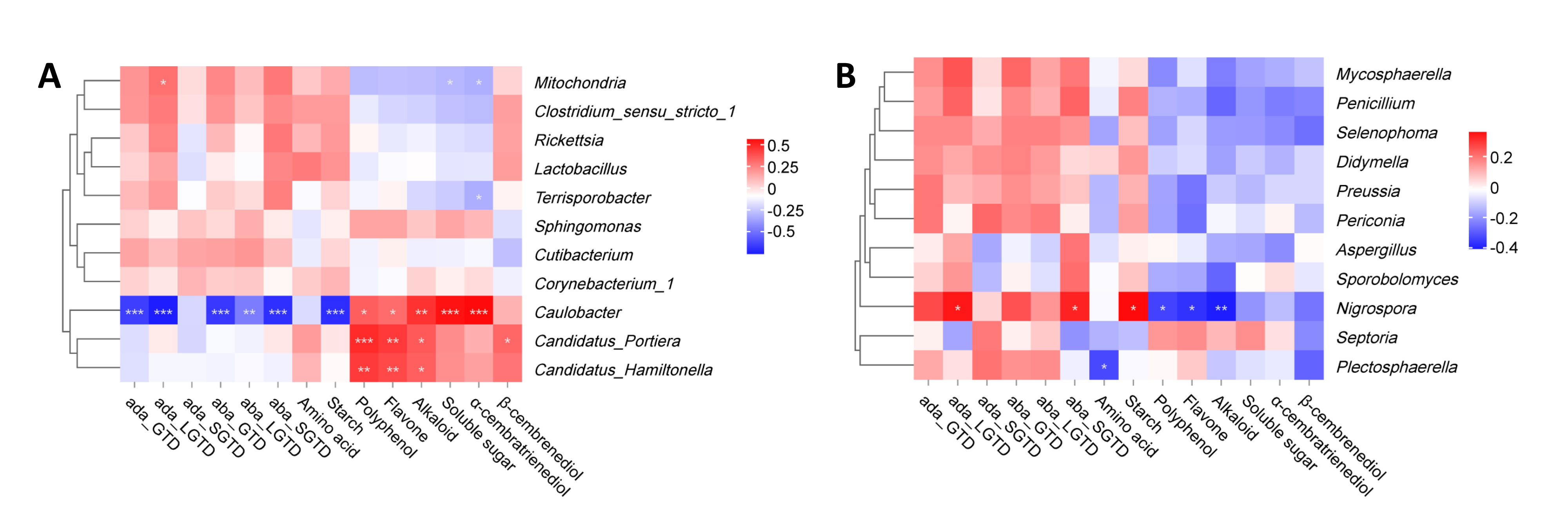

Supplement: Supplementary file 1 [file Image_1.tif]

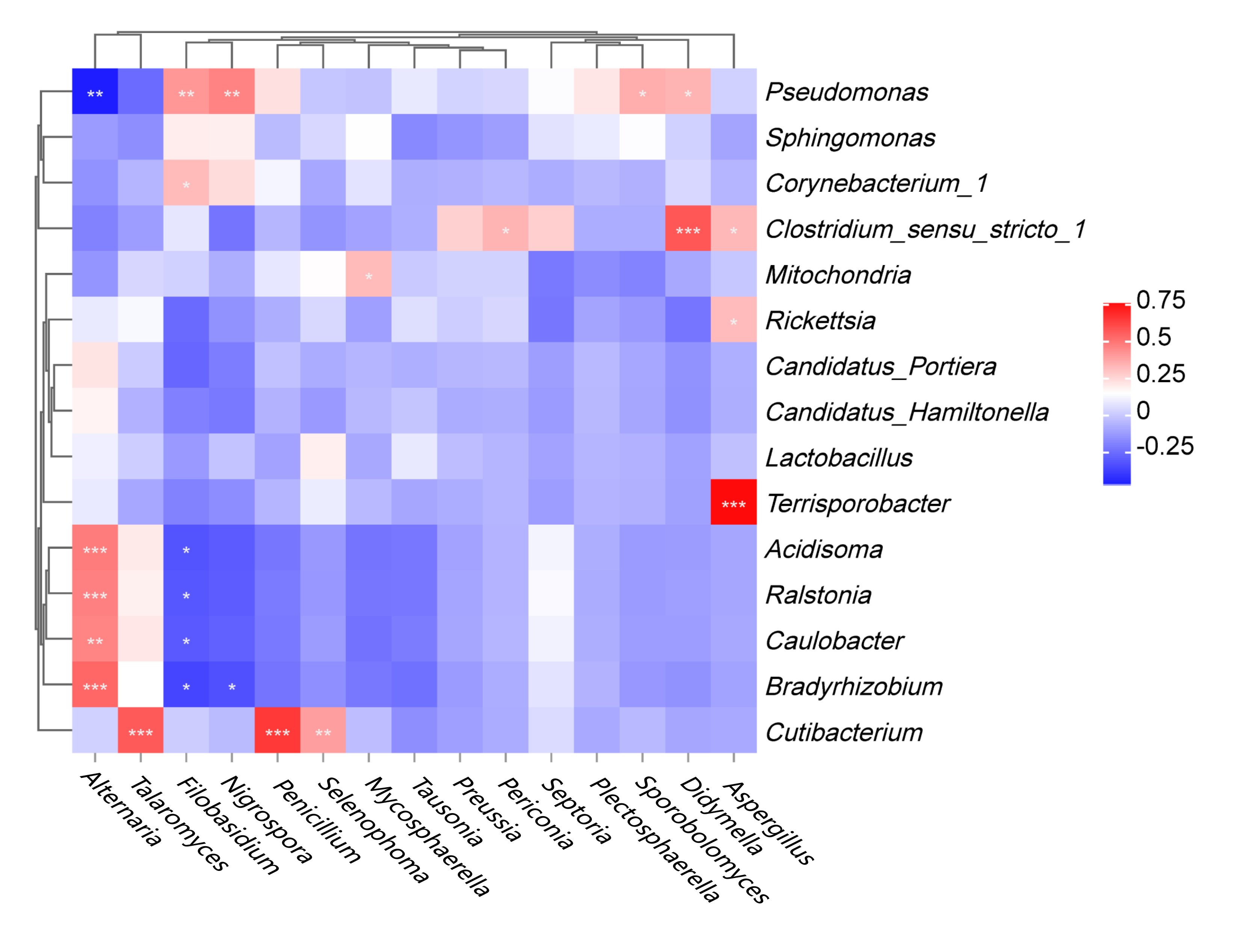

Supplement: Supplementary file 2 [file Image_2.tif]
